# Response of *Morus alba* L. to cadmium stress with potential for restoration: physiological and microbiological perspectives

**DOI:** 10.3389/fpls.2026.1795556

**Published:** 2026-04-22

**Authors:** Xiang Li, Dingping Zhao, Jiaheng Zhao, Chuang Li, Wen Deng, Shun Gao, Gang Chen, Hongling Hu

**Affiliations:** 1 Forest Ecology and Conservation in the Upper Reaches of the Yangtze River Key Laboratory of Sichuan Province, Chengdu, China; 2 Sichuan Mt. Emei Forest Ecosystem National Observation and Research Station, Sichuan Agricultural University, Chengdu, China

**Keywords:** cadmium tolerance, *Morus alba*, nutrient homeostasis, oxidative stress, rhizosphere microbiome

## Abstract

Cadmium (Cd) contamination threatens plant productivity and the stability of soil ecosystems. However, the mechanisms by which woody plants tolerate Cd stress remain incompletely understood. In this study, one-year-old *Morus alba* L. saplings were exposed to a gradient of Cd concentrations to investigate plant physiological responses, metal allocation patterns, cellular ultrastructure, and rhizosphere microbial communities. Increasing Cd concentrations progressively reduced plant growth, nutrient status, pigment content, and photosynthetic performance, while oxidative stress and membrane damage increased. In contrast, low Cd exposure induced a mild hormetic response, characterized by enhanced antioxidant activity, osmotic regulation, and a temporary increase in photosynthetic capacity. Bioaccumulation indices together with ultrastructural observations revealed a dose-dependent change in Cd handling. Under lower Cd exposure, Cd was more readily translocated to shoots, whereas higher Cd levels promoted root sequestration and intracellular compartmentalization. Despite Cd treatment, rhizosphere bacterial α-diversity remained relatively stable, although several Cd-tolerant taxa increased in relative abundance. Overall, these results demonstrate the tolerance capacity of *M. alba* to Cd stress and highlight its potential for the phytoremediation of mildly to moderately Cd-contaminated soils.

## 1 Introduction

Cadmium (Cd) contamination has become a major environmental concern due to its persistence, mobility, and toxicity in soil ecosystems. Anthropogenic activities, particularly industrial discharge and intensive agricultural practices, have significantly increased Cd accumulation in soils, posing risks to ecosystem stability and food security (Suhani et al., 2021; Yao et al., 2022). As a non-essential heavy metal, Cd disrupts a range of plant physiological processes, including oxidative balance, metabolic activity, and cellular growth, ultimately reducing plant productivity and ecological functioning (Khan et al., 2017). Consequently, identifying sustainable approaches for mitigating Cd contamination has become a major focus in soil restoration research.

Among the available remediation approaches, phytoremediation has received considerable attention as an environmentally friendly and cost-effective strategy for managing Cd-contaminated soils. This approach relies on plant physiological processes to extract, stabilize, or immobilize heavy metals within the soil–plant system (Wang and Aghajani Delavar, 2023). Compared with conventional physical and chemical remediation techniques, phytoremediation can also improve soil structure and ecosystem functioning, making it suitable for large-scale ecological restoration. However, many herbaceous species that exhibit high metal accumulation capacity are limited by relatively low biomass, shallow root systems, and short life cycles, which constrain their long-term remediation potential (Zulfiqar et al., 2022).

Woody plants, in contrast, generally develop deeper and more extensive root systems and produce greater biomass over longer growth periods. These characteristics enable them to access heavy metals from deeper soil layers and contribute to the long-term stabilization of contaminated environments (Song et al., 2024). Identifying woody species that combine strong Cd tolerance with effective metal accumulation capacity is therefore important for improving the efficiency and sustainability of phytoremediation strategies.

*Morus alba* L. (mulberry), a deciduous tree widely cultivated for both ecological restoration and economic production, has recently attracted attention as a potential species for phytoremediation. Previous studies have shown that *M. alba* can tolerate Cd stress through physiological adjustments, including enhanced antioxidant enzyme activities and structural modifications that reduce cellular damage (Guo et al., 2021; Zeng et al., 2020). In addition, mulberry has demonstrated the ability to absorb and accumulate Cd, suggesting potential applications in Cd-contaminated soils (Guo et al., 2021).

Recent studies have begun to clarify the mechanisms underlying Cd responses in *M. alba*. Transcriptomic analyses indicate that Cd exposure regulates genes associated with ion transport, antioxidant defense, hormone signaling, and cell wall biosynthesis (Dai et al., 2024). Experimental evidence also suggests that mulberry exhibits different tolerance responses across Cd concentrations, with variations in Cd accumulation patterns and physiological recovery depending on exposure intensity (Chen et al., 2025; Zeng et al., 2020). Metabolomic analyses further indicate that Cd stress can alter root exudate composition, potentially influencing both plant tolerance and rhizosphere interactions (Hu et al., 2023). Despite these advances, most existing studies have focused primarily on growth inhibition and oxidative stress responses. The impacts of Cd stress on photosynthetic processes, nutrient allocation, metal compartmentalization, and rhizosphere microbial dynamics remain less thoroughly investigated.

The rhizosphere microbiome also plays an important role in plant responses to environmental stress. Root-associated microorganisms can influence nutrient acquisition, modify metal bioavailability, and enhance plant tolerance to adverse conditions (Philippot et al., 2013). Some microbial taxa have been reported to improve Cd tolerance through mechanisms such as metal immobilization, siderophore production, and regulation of soil redox conditions (Luo et al., 2017). Although previous studies suggest that Cd stress may alter the rhizosphere microbial community associated with *M. alba* (Hu et al., 2023), the interactions between microbial community dynamics and plant physiological responses remain insufficiently understood.

To address these gaps, the present study investigated the responses of *M. alba* to a gradient of Cd exposure using a controlled pot experiment. Plant growth, photosynthetic characteristics, nutrient allocation, antioxidant responses, Cd accumulation and transport, root ultrastructure, and rhizosphere bacterial community composition were examined simultaneously. By integrating physiological, structural, and microbial perspectives, this study aims to improve understanding of Cd tolerance mechanisms in woody plants and to evaluate the potential of *M. alba* for the remediation of Cd-contaminated soils.

## 2 Materials and methods

### 2.1 Selection of plants

One-year-old *Morus alba* L. saplings were obtained from the Dechang County Forestry and Grassland Bureau of Liangshan Prefecture in November 2022. Saplings at this developmental stage were selected because they exhibit active growth and high physiological plasticity, which makes them suitable for evaluating plant responses to environmental stress under controlled conditions (Li et al., 2023). The use of uniform juvenile material also helped reduce ontogenetic variability among experimental individuals.

Saplings with similar morphology and vigorous growth were selected and transplanted into plastic pots containing a uniform growth substrate. Each pot contained a single sapling to avoid interplant competition. Prior to Cd treatment, plant height and basal diameter were measured, with an average plant height of 24.48 cm and an average basal diameter of 0.33 cm.

During the acclimation and cultivation period, soil moisture was monitored using an HH2 soil moisture meter (ML2x, GBR). Soil volumetric water content was maintained at approximately 30%, which represents moderate and non-limiting moisture conditions for woody saplings grown under pot culture (Turner, 2019). Maintaining this moisture level minimized drought or waterlogging effects, ensuring that observed plant responses were primarily associated with Cd treatments.

### 2.2 Experimental site

The experiment was conducted at the nursery base of Sichuan Agricultural University in Wenjiang District, Chengdu, Sichuan Province, China (103.8681° E, 30.7263° N; altitude 541 m). The region has a humid subtropical climate with distinct seasonal variation. The surrounding vegetation mainly consists of *Osmanthus fragrans*, *Cinnamomum japonicum*, and shrubs such as *Ligustrum lucidum* and *Rhododendron simsii*.

### 2.3 Experimental soil

The soil used in the experiment was collected from a riverside area along the Qingyi River in Ya’an City, Sichuan Province, China. Plant residues and stones were removed prior to use. The physicochemical properties of the soil were as follows: total N 2.24 g·kg^-^¹, total P 1.59 g·kg^-^¹, total K 6.34 g·kg^-^¹, Cd 0.26 mg·kg^-^¹, and pH 5.96.

### 2.4 Experimental design

A one-way completely randomized design was adopted. Based on Cd contamination levels commonly reported in Chinese agricultural soils, six Cd treatments were established: 0, 5, 10, 25, 50, and 100 mg·kg^-^¹, corresponding to CK, T1, T2, T3, T4, and T5, respectively. Each treatment included five replicate pots, resulting in a total of 30 experimental units. The selected Cd concentrations were based on reported contamination levels in agricultural soils and previous studies on Cd phytotoxicity (Violante et al., 2010; Zeng et al., 2020; Guo et al., 2021).

Cadmium was applied as CdCl_2_·2.5H_2_O dissolved in deionized water. To simulate gradual Cd input under natural conditions and reduce potential acute stress effects, the total Cd dose for each treatment was divided into three equal applications at 15-day intervals (Zhou et al., 2020). All pots were maintained in a ventilated greenhouse to prevent Cd loss caused by rainfall. Plastic trays were placed beneath the pots to collect leachate, which was returned to the soil when present. Pot positions were periodically rotated to minimize microenvironmental variation.

Cd treatments began in early April 2023. Plant height and basal diameter were measured before treatment application. Control plants received an equivalent volume of deionized water. At the end of the growing season in early November 2023, plants were harvested and separated into leaves, stems, trunks, and roots for biomass determination and subsequent analyses of nutrient elements and Cd concentrations.

Each pot containing a single sapling was treated as an independent experimental unit. The experiment lasted approximately 210 days (April–November 2023). For physiological and biochemical measurements, three technical replicates were conducted for each biological sample. Data are presented as mean ± standard error, and biological replicates were used for statistical analyses.

Each treatment consisted of five replicate pots (biological replicates). For physiological and biochemical analyses, three plants were randomly selected from the replicate pots of each treatment, and the measurements were conducted with three biological replicates (n = 3).

### 2.5 Growth indicators

(1) Plant height and basal diameter.

Plant height and basal diameter were measured in three randomly selected plants per treatment. Plant height was measured from the soil surface to the apex of the main stem using a measuring tape. Basal diameter was measured approximately 1 cm above the soil surface using a digital vernier caliper.

Net increases in plant height and basal diameter were calculated as the differences between the final and initial measurements during the Cd exposure period.

(2) Leaf area.

Leaf area was determined using ImageJ software (National Institutes of Health, USA). Digital images of leaves were analyzed, and leaf area was calculated using pixel conversion based on a reference area according to the following equation:


$$\text{Leaf area}=\text{reference area}\times \frac{\text{number of actual area pixels}}{\text{number of reference area pixels}}$$

(3) Biomass.

At harvest, three plants per treatment were randomly selected for biomass determination. Plants were carefully uprooted and separated into roots, stems, and leaves. Root systems were gently rinsed with deionized water to remove adhering soil particles. All samples were oven-dried at 80 °C to constant weight and then weighed to determine organ biomass.

### 2.6 Photosynthetic physiological indicators

#### 2.6.1 Chlorophyll content

Chlorophyll content was determined following the method described by Lichtenthaler and Wellburn (Lichtenthaler and Wellburn, 1983). The absorbance of the extract was measured at λ = 645, 663, and 470 nm using a Hach Lange DR-2800 spectrometer. Chlorophyll a, chlorophyll b, and carotenoid contents were calculated using the following equations.


Chla content (mg/g FW)=(12.72A663−2.59A645)×VT×FnW×100

Chlb content (mg/g FW)=(22.88A645−4.67A663)×VT×FnW×1000

Chl content (mg/g FW) = Chla content + Chlb content

#### 2.6.2 Light response curve

Light response curves were measured using a Li-6800 photosynthesis system. Light intensity gradients were set at 2100, 1800, 1500, 1200, 1000, 800, 600, 400, 200, 100, 50, 25, and 0 μmol·m^-^²·s^-^¹. The chamber temperature was maintained at 25 °C, relative humidity at 60%, and carbon dioxide concentration at 400 μmol/mol to simulate ambient atmospheric conditions.

(3) Light response characteristic parameters.

The following parameters were calculated from the model of Wang FB (Wang et al., 2023): AQY (Apparent quantum yield), Pmax (Maximum net photosynthetic rate), LSP (Light saturation point), LCP (Light compensation point), Rd (Dark respiration rate), and ELR (Effective Light Range).

### 2.7 Nutritional elements

Plant samples were oven-dried at 65–70 °C to constant weight and ground into fine powder prior to analysis. Nitrogen (N), phosphorus (P), and potassium (K) contents in different organs of *M. alba* were determined on a dry weight basis using the Kjeldahl nitrogen determination method (Scheiner, 1976), platinum antimony colorimetry (Mellon, 1959), and atomic absorption spectrophotometry (Hatch and Ott, 1968).

### 2.8 Antioxidant and osmotic regulation indices

Hydrogen peroxide (H_2_O_2_) and malondialdehyde (MDA) contents were determined using the ammonium molybdate–titanium colorimetric method and the thiobarbituric acid method, respectively. Fresh leaves (0.2–0.5 g) were homogenized in 5% trichloroacetic acid and centrifuged at 12, 000 r min^-^¹ for 20 min at 4 °C. The supernatant was used for analysis.

SOD, CAT, and POD activities were determined using the hydrazine method, ammonium molybdate method, and phenolphthalein colorimetric method, respectively. Enzyme extracts were prepared from fresh leaves homogenized in phosphate buffer (PBS, pH 7.8).

Soluble protein (SP), free proline (Pro), and soluble sugar (SS) contents were determined using the Coomassie Brilliant Blue method, acidic ninhydrin method, and phenol–sulfuric acid method, respectively.

### 2.9 Bioaccumulation factor and translocation factor

The bioaccumulation factor (BCF) was calculated as (McGrath and Zhao, 2003):


$$\text{BCF}=\frac{\text{Cd content in plants}}{\text{Cd content in soil}}$$

The translocation factor (TF) was calculated as (McGrath and Zhao, 2003):


$$\text{TF}=\frac{\text{Cd content in the above}-\text{ground part of the plant }}{\text{Cd content in the below}-\text{ground part of the plant}}$$

### 2.10 Rhizosphere microbial community of *M. alba*

Rhizosphere and bulk soils were collected following the method described by Bulgarelli (Bulgarelli et al., 2013). Soil tightly adhering to the root surface was considered rhizosphere soil, whereas soil collected away from the root system was regarded as bulk soil.

Total genomic DNA was extracted using the ZymoBIOMICS DNA Microprep Kit (Zymo Research, USA). The V4 region of the bacterial 16S rRNA gene was amplified using primers 515F (5′-GTGYCAGCMGCCGCGGTAA-3′) and 806R (5′-GGACTACHVGGGTWTCTAAT-3′). PCR amplification was performed under standard conditions with an initial denaturation at 95 °C for 3 min, followed by 25–30 cycles of denaturation at 95 °C for 30 s, annealing at 55 °C for 30 s, extension at 72 °C for 45 s, and a final extension at 72 °C for 5 min.

PCR products were purified, and sequencing libraries were constructed using the NEBNext Ultra II DNA Library Prep Kit. Libraries were sequenced on an Illumina NovaSeq 6000 platform.

Raw sequencing reads were quality-filtered and processed using QIIME2 (v2020.2). Amplicon sequence variants (ASVs) were inferred using the Deblur algorithm, with chimeric sequences removed during the process. Taxonomic classification of representative ASV sequences was performed using a Naive Bayes classifier trained against the SILVA database (v138).

Alpha diversity indices (Chao1, ACE, and Shannon) were calculated to assess bacterial richness and diversity within samples. Microbial community composition was evaluated based on the relative abundance of taxa at different taxonomic levels (e.g., phylum and genus). Functional profiles of microbial communities were predicted using PICRUSt2 based on the ASV feature table.

The raw sequencing data have been deposited in the NCBI Sequence Read Archive (SRA) under accession number PRJNA1439574.

### 2.11 Transmission electron microscopy observation

Root samples were fixed in glutaraldehyde, post-fixed in osmium tetroxide, dehydrated in a graded acetone series, and embedded in Epon 812 resin. Ultrathin sections were prepared using an ultramicrotome and stained with uranyl acetate and lead citrate. Ultrastructural observations were performed using a transmission electron microscope (JEM-1400FLASH, JEOL, Japan).

### 2.12 Data analysis and visualization

Experimental data were processed using Microsoft Excel 2019. Statistical analyses were performed using SPSS 26.0 (IBM, USA). Differences among treatments were evaluated using one-way analysis of variance (ANOVA) followed by Duncan’s multiple range test (P< 0.05). Graphs were generated using Origin 2021. Principal component analysis (PCA) and Mantel tests were used to examine relationships between Cd allocation and physiological traits.

## 3 Results

### 3.1 Effects of Cd on plant growth

**Figure 1:**
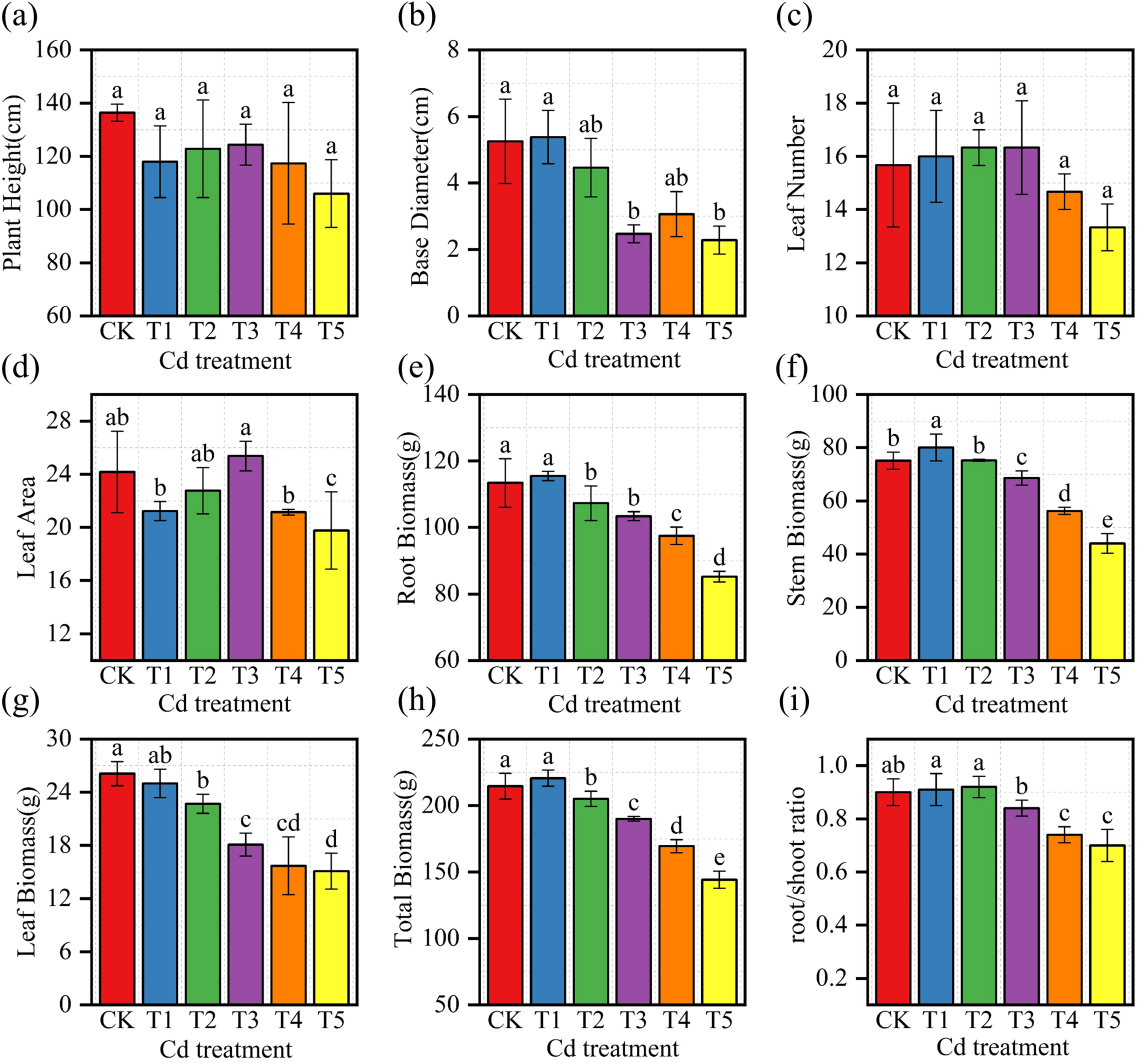
Effects of Cd treatments on growth traits of *M. alba* saplings. **(a)** Plant height; **(b)** Basal diameter; **(c)** Leaf number; **(d)** Leaf area; **(e)** Root biomass; **(f)** Stem biomass; **(g)** Leaf biomass; **(h)** Total biomass; **(i)** Root-to-shoot ratio Values are presented as mean ± SD (n = 3). Different letters indicate significant differences among treatments based on one-way ANOVA followed by Duncan’s multiple range test (P 0.05).

Plant growth and biomass were significantly affected by Cd treatments (**Figure 1**). Plant height, basal diameter, and total biomass generally declined with increasing Cd concentration. Leaf number in T3 (16.33 ± 1.76) was slightly higher than that in CK (15.67 ± 2.33), although the difference was not significant (P > 0.05). Stem biomass increased from 75.1 ± 3.23 g in CK to 80.1 ± 5.05 g in T1 (P< 0.05), and total biomass reached its highest value in T1 (220.6 ± 6.09 g), which was significantly greater than that in CK (P< 0.05). At higher Cd concentrations (T4–T5), root and leaf biomass decreased significantly compared with CK (P< 0.05).

### 3.2 Effect of Cd on photosynthesis

**Figure 2:**
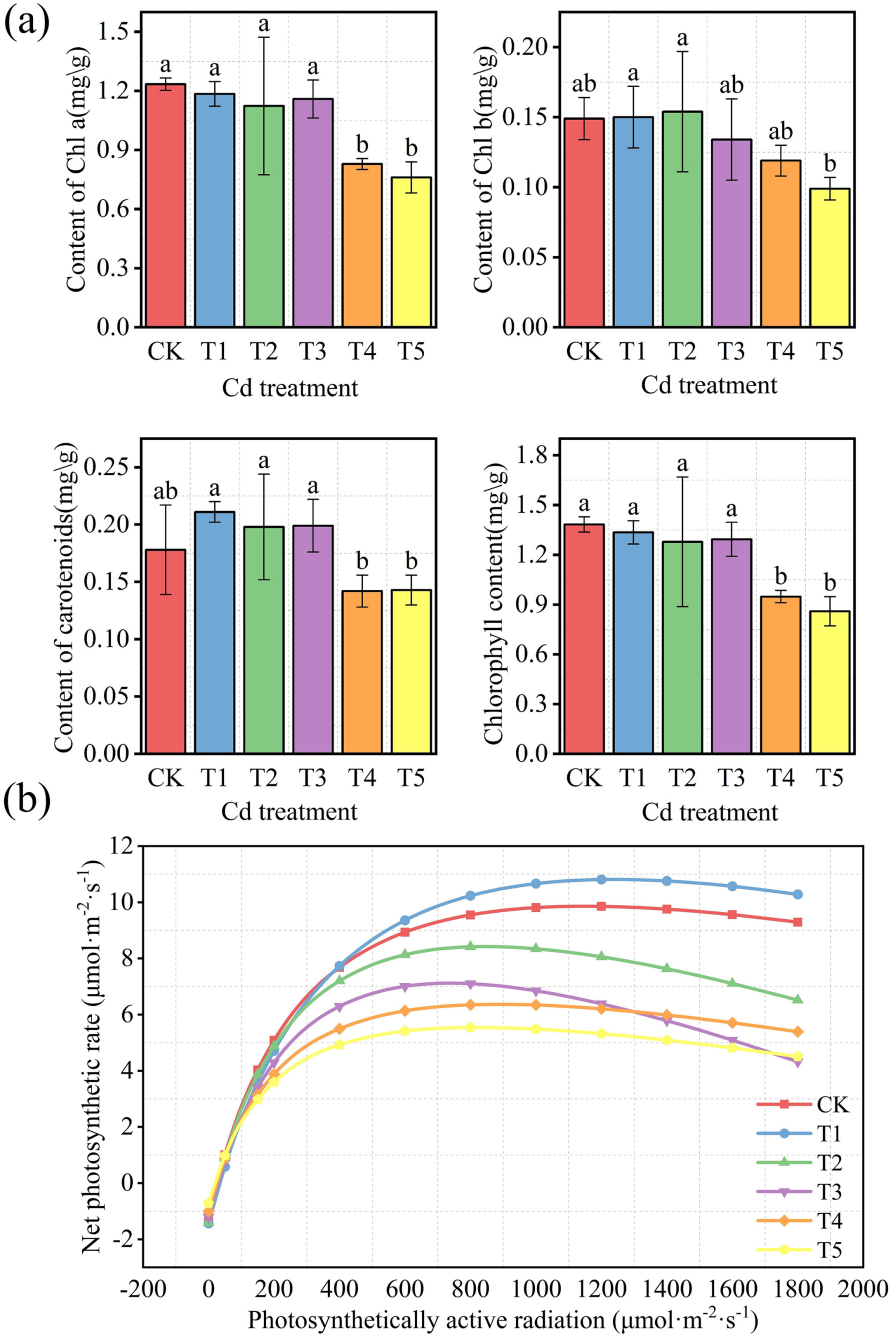
Effects of Cd treatments on photosynthetic pigments and light response characteristics of *M. alba*. **(a)** Chlorophyll a, chlorophyll b, carotenoids, and total chlorophyll contents. **(b)** Light response curves of net photosynthetic rate under different Cd treatments. Values in panel **(a)** are presented as mean ± SD (n = 3). Different letters indicate significant differences among treatments based on one-way ANOVA followed by Duncan’s multiple range test (P< 0.05).

Photosynthetic pigment contents were significantly affected by Cd treatment (**Figure 2a**). Chlorophyll a, chlorophyll b, carotenoid, and total chlorophyll contents decreased progressively with increasing Cd concentration. In particular, chlorophyll a, carotenoid, and total chlorophyll contents in T4 and T5 were significantly lower than those in CK (P< 0.05).

Light response curves showed similar overall patterns among treatments (**Figure 2b**). Net photosynthetic rate increased rapidly under low photosynthetically active radiation (PAR), approached a maximum at intermediate light intensities, and then declined slightly at higher radiation levels. The maximum net photosynthetic rate occurred within the range of 700–1200 μmol·m^-^²·s^-^¹, and the treatments were ranked as T1 > CK > T2 > T3 > T4 > T5. Among them, T1 (5 mg·kg^-^¹ Cd) showed the highest Pmax, whereas higher Cd concentrations were associated with progressive declines.

As shown in **Table 1**, AQY values did not differ significantly among treatments (P > 0.05). In contrast, Pmax varied markedly with Cd concentration, with T1 exhibiting the highest value, which was significantly greater than those of the other treatments (P< 0.05), whereas T4 and T5 showed the lowest values. Similarly, LSP reached its maximum in T1 and was significantly higher than in the remaining treatments (P< 0.05). By comparison, LCP and Rd showed no significant differences among treatments (P > 0.05). ELR displayed a pattern similar to that of Pmax, with T1 showing the highest value (P< 0.05).

**Table 1:** Characteristic parameters of light response of saplings at different Cd levels.

Cd Treatment	AQY	P_max_	LSP	LCP	Rd	ELR
CK	0.049 ± 0.007a	8.55 ± 0.72b	1026.36 ± 92.3ab	34.23 ± 7.12a	1.46 ± 0.15a	992.13 ± 91.2ab
T1	0.047 ± 0.004a	10.49 ± 0.32a	1209.20 ± 29.8a	31.41 ± 2.89a	1.36 ± 0.07a	1177.79 ± 26.9a
T2	0.047 ± 0.008a	7.30 ± 0.56bc	1072.08 ± 107.1ab	23.63 ± 2.64a	1.02 ± 0.20a	1048.45 ± 117.5ab
T3	0.046 ± 0.002a	6.36 ± 0.41c	914.55 ± 96.5b	30.61 ± 4.61a	1.24 ± 0.13a	883.94 ± 93.8b
T4	0.049 ± 0.005a	6.15 ± 0.63c	829.77 ± 45.6b	23.24 ± 3.51a	1.01 ± 0.12a	806.53 ± 48.1b
T5	0.045 ± 0.005a	4.51 ± 0.55d	827.23 ± 27.5b	32.01 ± 6.91a	1.19 ± 0.26a	795.21 ± 29.5b

Values are presented as mean ± SD (n = 3). Different letters indicate significant differences among treatments based on one-way ANOVA followed by Duncan’s multiple range test (P< 0.05).

### 3.3 Effect of Cd on nutrients

**Figure 3:**
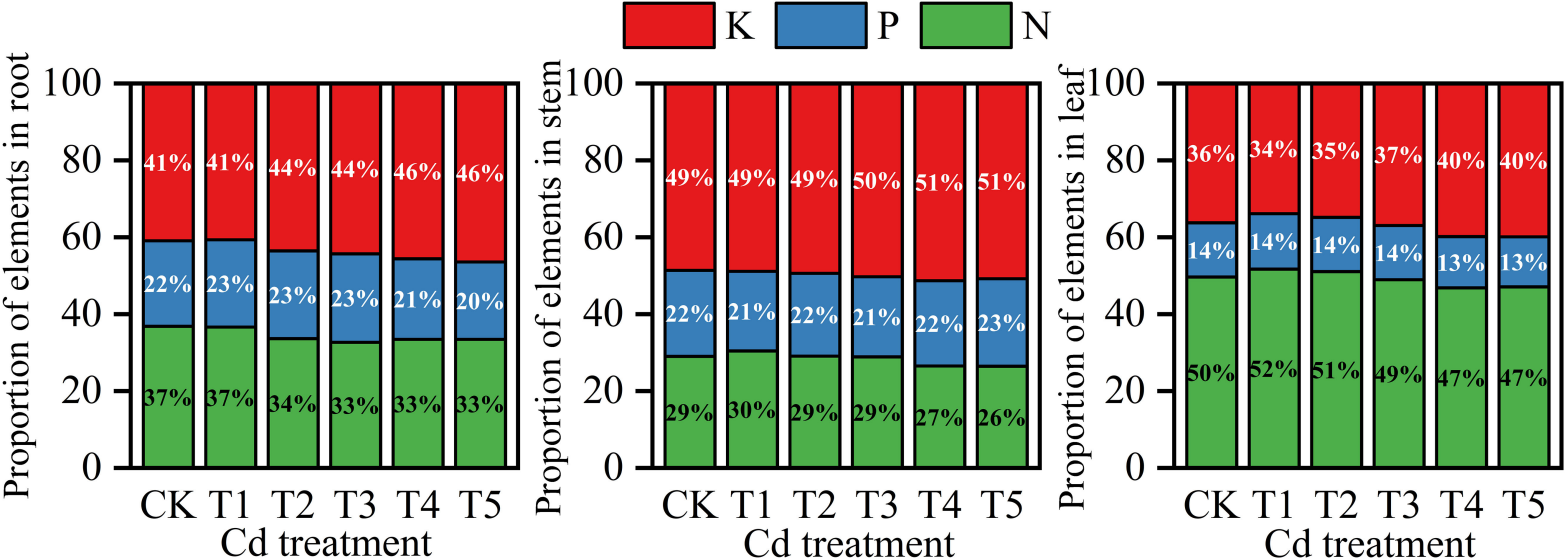
Proportional distribution of N, P, and K in roots, stems, and leaves of *M. alba* under different Cd treatments.

Elemental proportions differed among plant organs under Cd treatment (**Figure 3**). In roots, the proportions of N and P gradually decreased with increasing Cd concentration, whereas the proportion of K increased correspondingly. In stems, the proportions of K and P remained relatively stable across treatments, while the proportion of N showed a slight decline at higher Cd levels. In leaves, the proportion of P exhibited minimal variation, whereas the proportion of N increased slightly at low Cd levels and then declined under higher Cd treatments.

Cd exposure significantly influenced nutrient contents in different plant organs (**Table 2**). In roots, N and P contents declined progressively with increasing Cd concentration, whereas K content showed a slight decrease but remained relatively stable compared with other nutrients. In stems, N, P, and K contents all decreased with increasing Cd levels, with N showing the most pronounced reduction. In leaves, nutrient contents were highest in the control treatment and declined gradually along the Cd gradient, particularly for N and P.

**Table 2:** Nutrient content of saplings at different Cd levels.

Nutrient elements	Plant organs	Cd treatment					
		CK	T1	T2	T3	T4	T5
N (g/kg)	Root	2.07 ± 0.05a	1.92 ± 0.04b	1.64 ± 0.05c	1.52 ± 0.03d	1.47 ± 0.01d	1.45 ± 0.02d
	Stem	1.25 ± 0.03a	1.19 ± 0.01b	1.11 ± 0.03c	1.07 ± 0.04c	0.92 ± 0.03d	0.87 ± 0.02e
	Leaf	5.06 ± 0.03a	4.83 ± 0.09b	4.35 ± 0.06c	3.93 ± 0.04d	3.37 ± 0.14e	2.97 ± 0.05f
P (g/kg)	Root	1.21 ± 0.03a	1.16 ± 0.01b	1.05 ± 0.03c	0.97 ± 0.04c	0.89 ± 0.03d	0.81 ± 0.02e
	Stem	0.96 ± 0.02a	0.81 ± 0.03b	0.82 ± 0.06b	0.77 ± 0.05bc	0.77 ± 0.04bc	0.75 ± 0.03c
	Leaf	1.43 ± 0.02a	1.35 ± 0.03b	1.20 ± 0.06c	1.13 ± 0.05d	0.96 ± 0.04e	0.82 ± 0.03f
K (g/kg)	Root	2.30 ± 0.06a	2.13 ± 0.20ab	2.12 ± 0.13ab	2.06 ± 0.12b	2.00 ± 0.16b	2.01 ± 0.11b
	Stem	2.09 ± 0.11a	1.91 ± 0.08b	1.88 ± 0.09b	1.86 ± 0.09b	1.78 ± 0.03bc	1.67 ± 0.11c
	Leaf	3.69 ± 0.40a	3.16 ± 0.18b	2.96 ± 0.10b	2.96 ± 0.14b	2.86 ± 0.28b	2.51 ± 0.10c

Values are presented as mean ± SD (n = 3). Different letters indicate significant differences among treatments based on one-way ANOVA followed by Duncan’s multiple range test (P< 0.05).

### 3.4 Effects of Cd on the antioxidant and osmoregulatory systems

**Figure 4:**
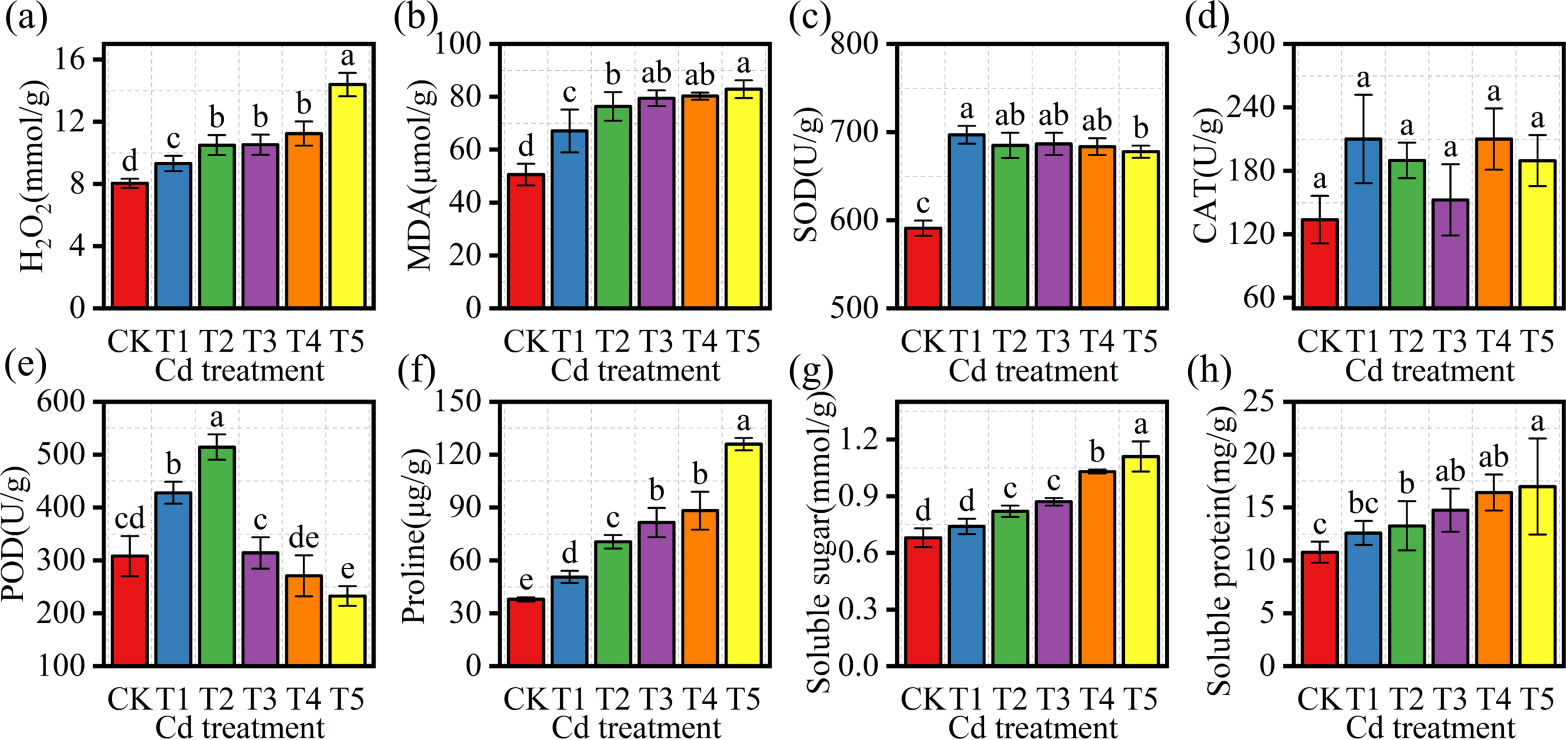
Antioxidant enzyme activities and osmoregulatory compounds in *M. alba* under Cd treatments. **(a)** H_2_O_2_ content; **(b)** MDA content; **(c)** SOD activity; **(d)** CAT activity; **(e)** POD activity; **(f)** proline content; **(g)** soluble sugar content; **(h)** soluble protein content. Values are presented as mean ± SD (n = 3). Different letters indicate significant differences among treatments based on one-way ANOVA followed by Duncan’s multiple range test (P< 0.05).

H_2_O_2_ and MDA contents increased with rising Cd concentration (**Figures 4a, b**). Both indicators reached their highest levels in T5 (14.39 ± 0.75 and 82.95 ± 0.36, respectively), which were significantly higher than those observed in the other treatments (P< 0.05). Antioxidant enzyme activities showed distinct patterns among treatments (**Figures 4c–e**). SOD activity increased under low Cd exposure and peaked in T1 (696.88 ± 10.06), then declined gradually at higher Cd concentrations, although all Cd treatments maintained significantly higher SOD activity than CK (P< 0.05). CAT activity varied among treatments, with comparatively higher values in T1 and T4. POD activity increased at lower Cd levels and reached its maximum in T2 (514.33 ± 23.86), which was significantly higher than in the other treatments (P< 0.05), before declining under higher Cd exposure. Osmoregulatory substances, including proline (Pro), soluble sugars (SS), and soluble protein (SP), increased along the Cd gradient and reached their highest levels in T5, all significantly higher than CK (P< 0.05) (**Figures 4f–h**).

### 3.5 Cd accumulation and translocation in *M. alba*

BCF values of roots, stems, leaves, and aboveground tissues declined significantly with increasing Cd concentration (P< 0.05) (**Table 3**). In T1, the BCF values of roots and leaves exceeded 1, whereas values in all other treatments remained below 1. The translocation factor (TF) showed a unimodal response to Cd exposure, increasing under low to moderate Cd levels and reaching its maximum in T3, where it was significantly higher than in CK (P< 0.05). Relative to CK, TF values in T1–T5 increased by 182.23%, 243.51%, 433.26%, 182.30%, and 207.87%, respectively, and remained greater than 1 across all Cd treatments.

**Table 3:** Bioaccumulation factor and translocation factor of saplings at different Cd levels.

Cd treatment	Root BCF	Stem BCF	Leaf BCF	Aboveground BCF	TF
CK	—	—	—	—	0.47 ± 0.07b
T1	1.14 ± 0.21a	0.32 ± 0.10a	1.07 ± 0.39a	1.39 ± 0.49a	1.33 ± 0.54ab
T2	0.33 ± 0.08b	0.07 ± 0.01b	0.51 ± 0.29ab	0.58 ± 0.29b	1.61 ± 0.67ab
T3	0.12 ± 0.03c	0.05 ± 0.01b	0.25 ± 0.03b	0.30 ± 0.04b	2.51 ± 0.33a
T4	0.06 ± 0.02c	0.02 ± 0.01b	0.06 ± 0.02b	0.08 ± 0.02b	1.33 ± 0.26ab
T5	0.06 ± 0.03c	0.02 ± 0.01b	0.06 ± 0.02b	0.08 ± 0.03b	1.45 ± 0.51ab

Values are presented as mean ± SD (n = 3). Different letters indicate significant differences among treatments based on one-way ANOVA followed by Duncan’s multiple range test (P< 0.05). “—” indicates not applicable (CK contains no added Cd).

### 3.6 Ultrastructure of plants

**Figure 5:**
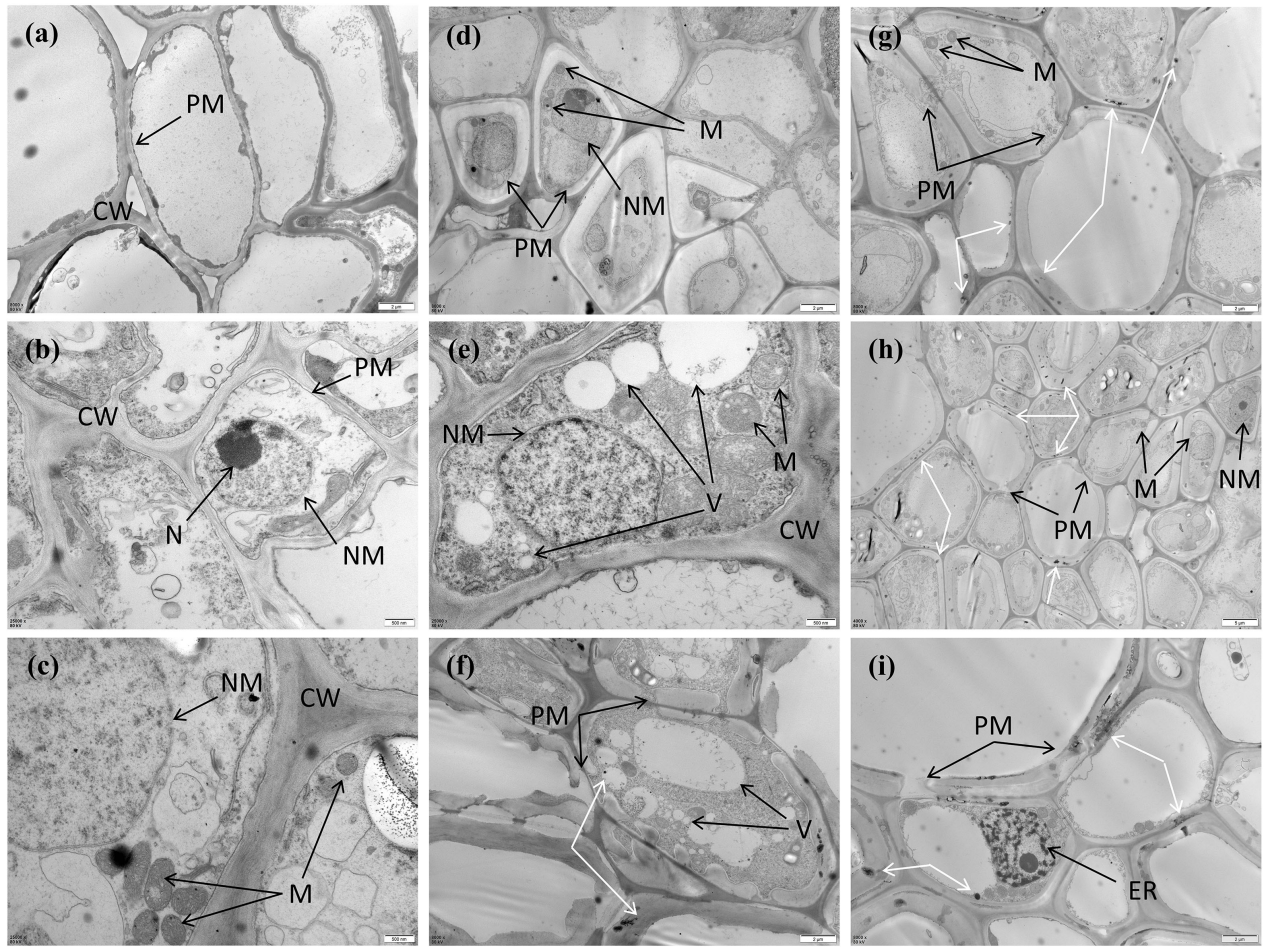
Transmission electron microscopy (TEM) images of root cells of *M. alba* under different Cd treatments. Representative ultrastructural features are shown for **(a–c)** CK, **(d–f)** T3, and **(g–i)** T5 treatments. CW, cell wall; PM, plasma membrane; M, mitochondria; V, vesicles; NM, nuclear membrane; N, nucleus; ER, endoplasmic reticulum. White arrows indicate electron-dense deposits. Scale bars are shown in each panel.

Ultrastructural characteristics differed among Cd treatments as observed by transmission electron microscopy (**Figure 5**). In the CK group, cells exhibited a thick and intact cell wall, a continuous plasma membrane, a clearly defined nucleolus, and oval mitochondria evenly distributed within the cytoplasm (**Figures 5b, c**). Under the T3 treatment, electron-dense granules accumulated along the cell wall and vesicle margins (**Figure 5f**). Partial disruption of the plasma membrane and swelling of mitochondrial cristae were evident (**Figure 5e**), together with an increased number of vesicles in some cells (**Figure 5f**). In the T5 treatment, structural damage became more pronounced, including rupture of the cell wall (**Figure 5i**) and substantial accumulation of electron-dense deposits within the cell wall and vesicles (**Figure 5h**). In addition, metal-like particulate structures appeared in certain cells.

### 3.7 Rhizosphere microbial communities of *M. alba* under Cd stress

#### 3.7.1 Bacterial community α-diversity analysis

**Figure 6:**
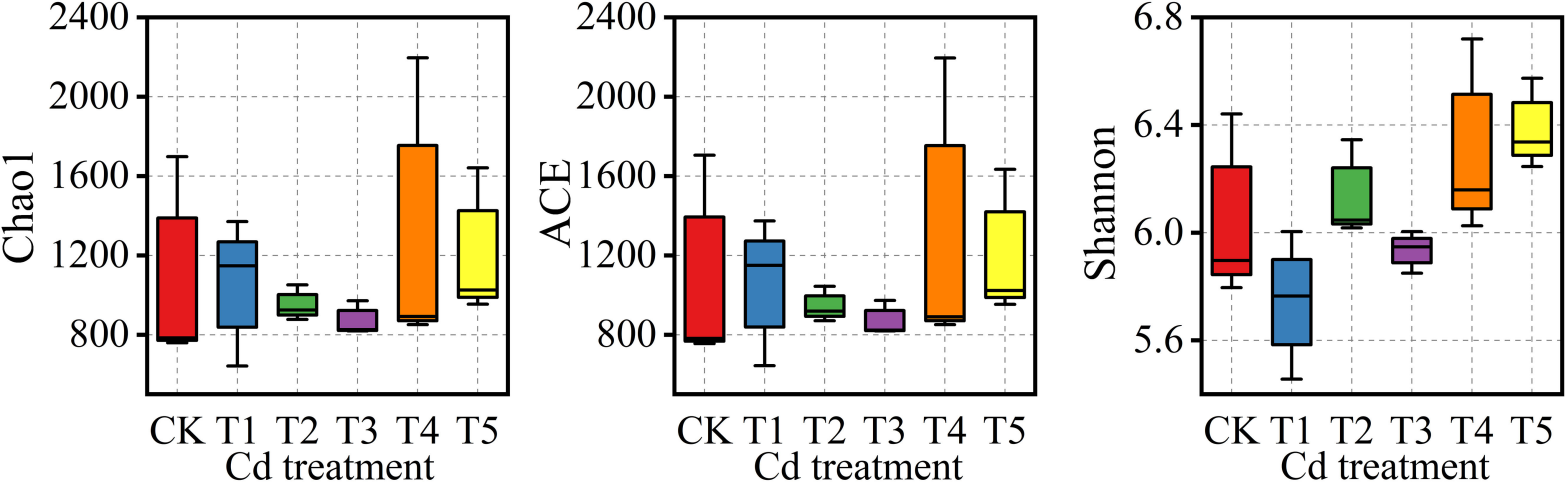
Alpha diversity of rhizosphere bacterial communities under different Cd treatments. Boxplots represent the Chao1, ACE, and Shannon diversity indices across treatments (CK–T5). Boxes indicate the interquartile range (IQR), horizontal lines represent medians, and whiskers denote minimum and maximum values.

Alpha-diversity indices remained relatively stable across Cd treatments (**Figure 6**). The Chao1 index ranged from 871 to 1312 among CK–T5, with values of 1079, 1053, 950, 871, 1312, and 1206, respectively. The Shannon index varied between 5.74 and 6.39, whereas the Simpson index ranged from 0.9884 to 0.9967. Statistical analysis showed no significant differences among treatments (P > 0.05).

#### 3.7.2 Soil bacterial community structure analysis

**Figure 7:**
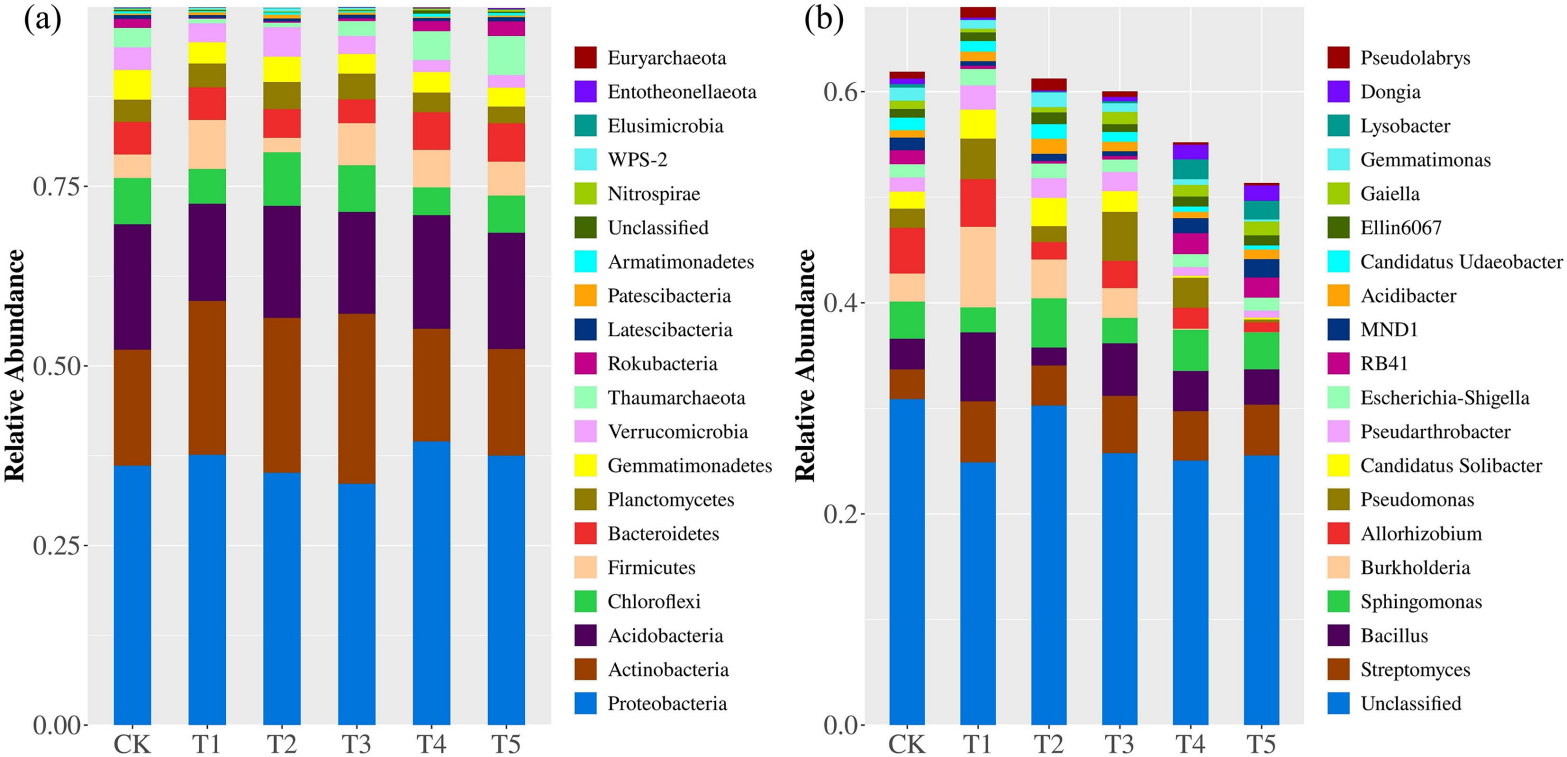
Relative abundance of rhizosphere bacterial communities under different Cd treatments. Taxonomic composition is shown at the **(a)** phylum level and **(b)** genus level. Bars represent the relative abundance of dominant taxa across treatments (CK–T5). Only major taxa are displayed, and low-abundance groups are grouped as “Unclassified” where applicable.

Cd treatments influenced the composition of the rhizosphere bacterial community while the overall community structure remained broadly stable (**Figure 7**). At the phylum level, Proteobacteria remained dominant across all treatments. The relative abundances of Actinobacteria, Acidobacteria, Chloroflexi, Bacteroidetes, and Firmicutes varied among Cd treatments and showed fluctuations along the Cd gradient, although no phylum was completely lost or replaced. At the genus level, variation was more pronounced. Unclassified taxa maintained relatively high abundance across treatments, whereas several genera decreased with increasing Cd concentration. In contrast, *Bacillus*, *Sphingomonas*, *Burkholderia*, and *Pseudomonas* showed higher relative abundances under medium to high Cd levels.

#### 3.7.3 Soil bacterial community functional prediction analysis

**Figure 8:**
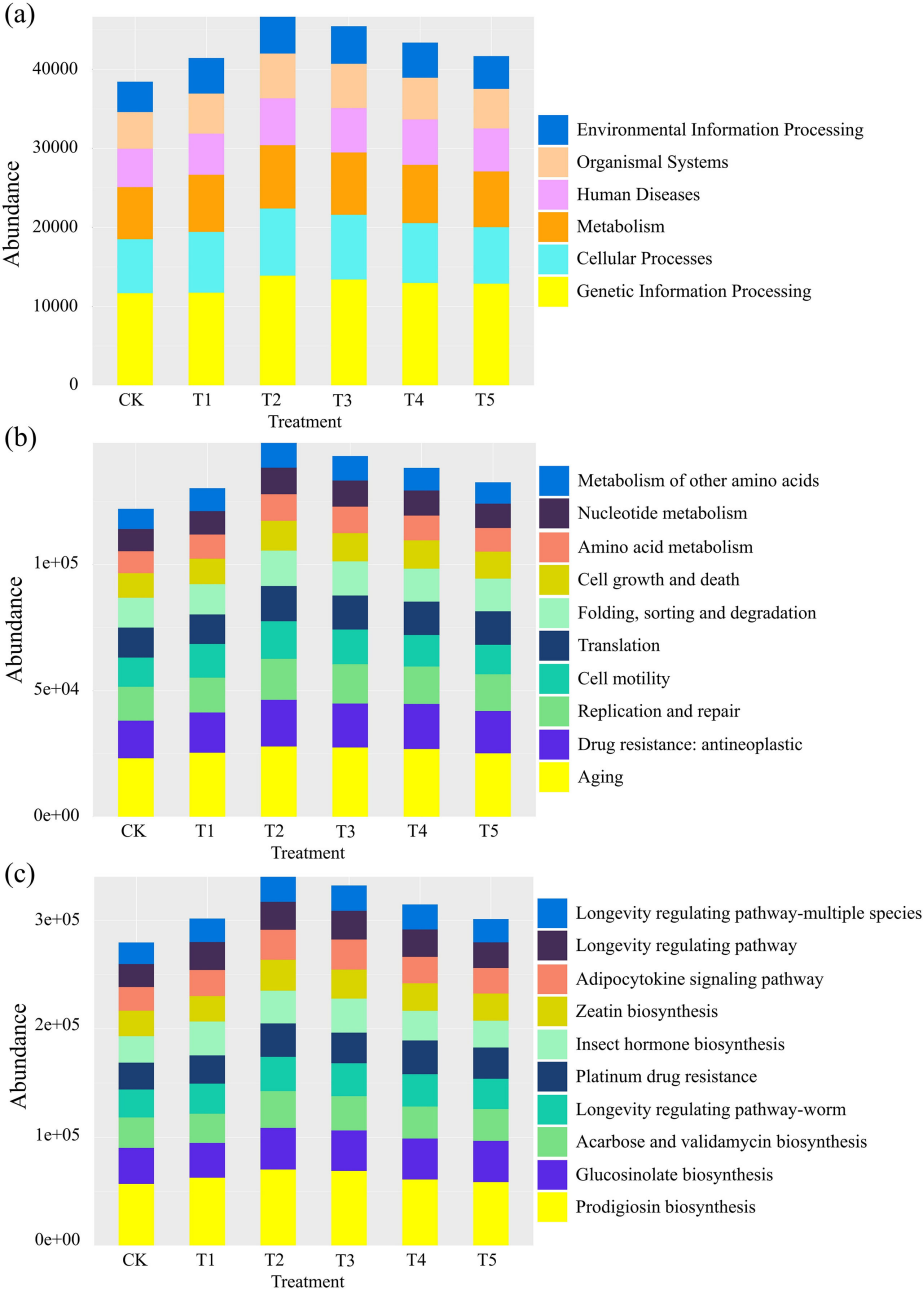
Functional prediction of rhizosphere bacterial communities in *M. alba* based on PICRUSt2 analysis. Relative abundances of predicted KEGG Orthology (KO) pathways at three hierarchical levels are shown: **(a)** Level 1 functional categories, **(b)** Level 2 functional pathways, and **(c)** Level 3 functional pathways. Functional profiles were inferred from 16S rRNA gene sequences using PICRUSt2. Treatments correspond to increasing Cd concentrations (CK–T5).

Functional profiles of the microbial communities were predicted using PICRUSt2 and summarized across three hierarchical levels of KEGG Orthology (KO) metabolic pathways (**Figure 8**). At the first level (**Figure 8a**), genes associated with Genetic Information Processing were the most abundant across all treatments, followed by those related to Cellular Processes and Metabolism. At the second level (**Figure 8b**), the ten most abundant functional categories were similar among treatments, including Aging, Drug resistance/antineoplastic, Replication and repair, Cell motility, Translation, Folding, sorting and degradation, Cell growth and death, Amino acid metabolism, Nucleotide metabolism, and Metabolism of other amino acids. At the third level (**Figure 8c**), functional gene abundances remained generally comparable among treatments. The ten most abundant pathways included Prodigiosin biosynthesis, Glucosinolate biosynthesis, Acarbose and validamycin biosynthesis, Longevity regulating pathway–worm, Platinum drug resistance, Insect hormone biosynthesis, Zeatin biosynthesis, Adipocytokine signaling pathway, Longevity regulating pathway, and Longevity regulating pathway–multiple species. Despite the overall similarity in functional composition, relative abundances of several pathways varied among treatments, with genes related to Prodigiosin biosynthesis and Glucosinolate biosynthesis showing higher abundance in T2 and T3 than in CK.

### 3.8 Multivariate data analysis

#### 3.8.1 Principal component analysis of Cd-induced physiological responses

**Figure 9:**
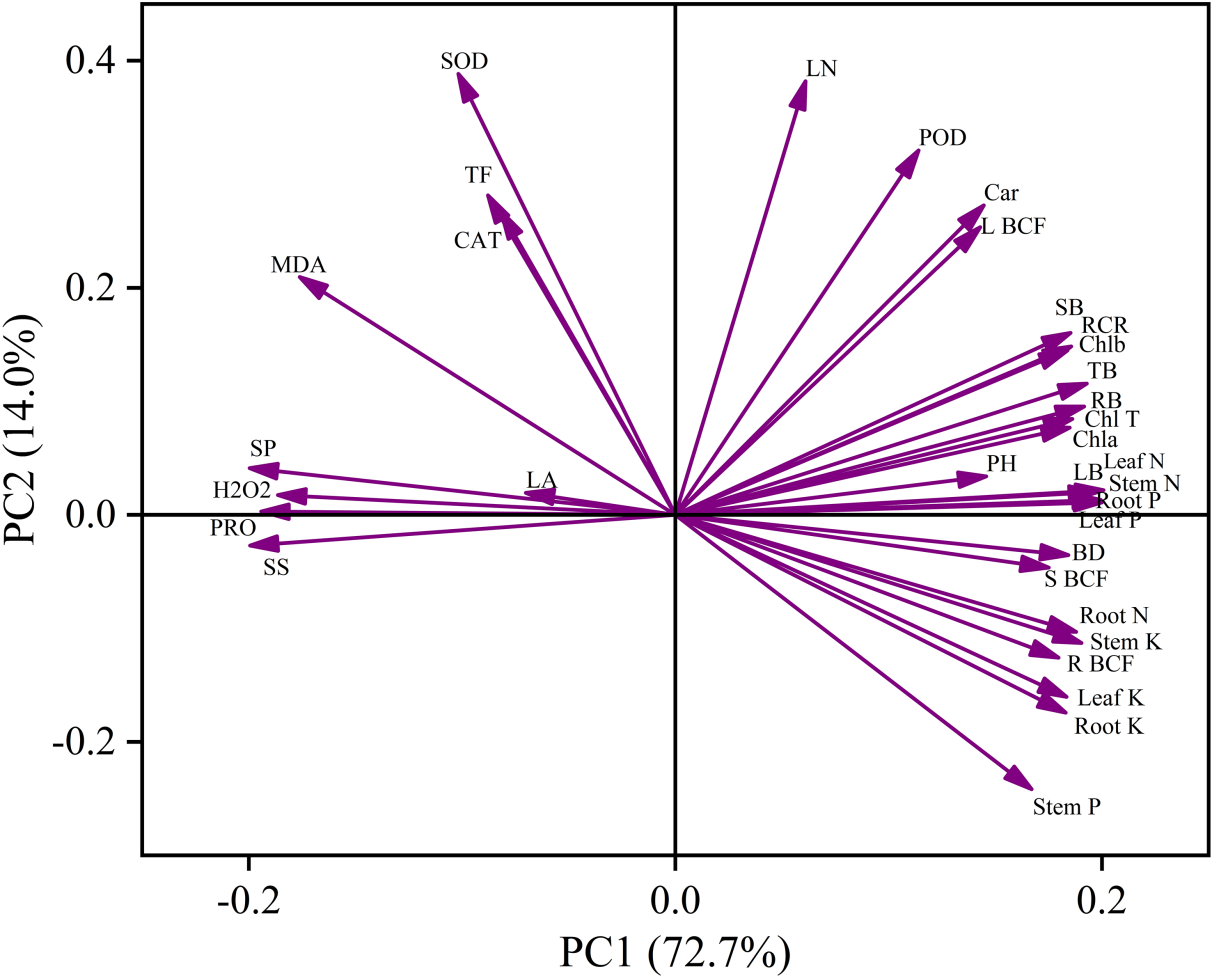
Principal component analysis (PCA) of Cd-induced physiological traits. The first two principal components (PC1 and PC2) explained 72.7% and 14.0% of the total variance, respectively. Arrows represent variable loadings, with vector length indicating the contribution of each trait to the principal components and direction reflecting correlation patterns among variables. Traits positioned in the same direction are positively correlated, whereas those in opposite directions are negatively associated. PH, plant height; BD, basal diameter; LN, leaf number; RB, root biomass; SB, stem biomass; LB, leaf biomass; TB, total biomass; RCR, root-to-shoot ratio.

Principal component analysis (PCA) showed distinct multivariate patterns in plant physiological responses to Cd stress (**Figure 9**). The first two principal components explained 86.7% of the total variance, with PC1 accounting for 72.7% and PC2 for 14.0%. PC1 was mainly associated with growth, photosynthetic performance, and nutrient status, displaying positive loadings for biomass traits, photosynthetic pigments, nutrient contents, and bioconcentration factors. In contrast, oxidative stress indicators (MDA, H_2_O_2_, soluble sugars, soluble protein, and proline) loaded negatively on this axis. PC2 was primarily characterized by antioxidant enzyme activities and Cd translocation, with strong positive loadings for SOD, POD, CAT, and the translocation factor (TF). Growth- and metabolism-related traits were therefore largely aligned with PC1, whereas antioxidant regulation was more strongly associated with PC2.

#### 3.8.2 Mantel test reveals the association between Cd allocation and physiological traits

**Figure 10:**
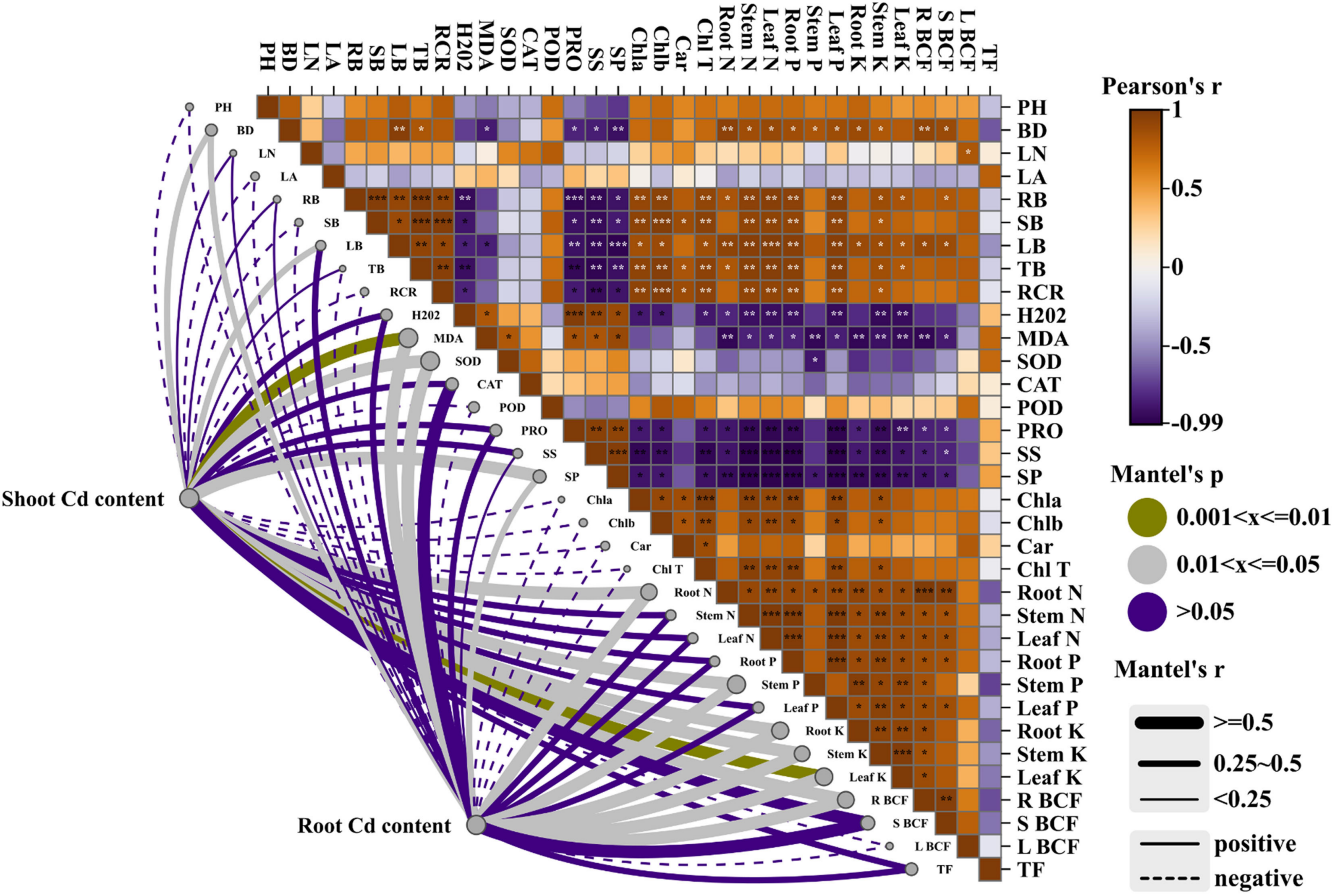
Mantel test and Pearson correlation analysis of associations between Cd allocation and plant physiological traits. The upper panel shows the Pearson correlation matrix among physiological variables, with color gradients representing correlation coefficients (r). The lower panel illustrates Mantel correlations between root and shoot Cd content and plant functional traits. Line thickness indicates Mantel’s r value, line color represents significance levels (Mantel’s p), and solid or dashed lines denote positive or negative correlations, respectively. Abbreviations are the same as in **Figure 9**.

Mantel test analysis identified contrasting associations between Cd accumulation in different plant organs and plant physiological traits (**Figure 10**). Root Cd content showed more frequent and stronger correlations with plant functional traits than shoot Cd content. Significant correlations were observed between root Cd content and biomass traits, antioxidant enzyme activities, oxidative stress indicators, nutrient contents, and Cd accumulation parameters. In comparison, shoot Cd content showed fewer and generally weaker correlations with these trait groups. Traits related to plant growth, photosynthetic pigments, and nutrient status were more closely associated with root Cd content, whereas stress-related variables exhibited weaker or inconsistent correlations with shoot Cd content.

## 4 Discussion

### 4.1 Growth restriction and hormetic stimulation under Cd exposure

Multivariate analysis identified root Cd accumulation as an important factor shaping plant responses to Cd stress. Against this background, the observed changes in growth, photosynthesis, biomass allocation, and rhizosphere characteristics can be understood as interconnected responses rather than independent effects.

Cd exposure had a clear inhibitory effect on the growth of *M. alba*. Plant height, basal diameter, and total biomass declined progressively with increasing Cd concentration (**Figures 1a–h**). Total biomass was significantly lower in T4 and T5 than in CK (P< 0.05; **Figure 1h**), and both root and leaf biomass decreased markedly when Cd levels exceeded 25 mg·kg^-^¹ (**Figures 1e, g**). At the same time, the root-to-shoot ratio increased under higher Cd treatments (**Figure 1i**), indicating a shift in biomass allocation toward belowground tissues. Similar growth suppression under elevated Cd exposure has been reported in other woody plants and is commonly associated with disruption of membrane integrity, ion transport, and cell division (Carvalho et al., 2020; Dubey et al., 2018; Saini et al., 2021).

A different response was observed at low Cd exposure. Plants treated with T1 (5 mg·kg^-^¹) showed significantly higher stem biomass and total biomass than CK (P< 0.05; **Figures 1f, h**), indicating a stimulatory effect under sub-toxic conditions. Although leaf number and leaf area in T3 did not differ significantly from CK (**Figures 1c, d**), the biomass increase at T1 supports the presence of a hormetic response. Comparable biphasic growth patterns have been reported in several plant species, where mild stress stimulates compensatory metabolic activity and temporarily promotes growth (Li et al., 2019; Zhang et al., 2020; Zhao et al., 2021).

The overall pattern suggests that *M. alba* maintains relatively stable growth under low Cd exposure but shows marked suppression once Cd concentrations exceed 25 mg·kg^-^¹, pointing to a physiological tolerance threshold. The coexistence of low-dose stimulation and high-dose inhibition reflects a balance between metabolic disturbance caused by Cd and the plant’s compensatory allocation of resources.

Similar biphasic responses have also been described in other woody species exposed to mild Cd stress (Carvalho et al., 2020; Zhao et al., 2021). In comparison with many herbaceous hyperaccumulators, which often exhibit rapid growth inhibition even at moderate Cd levels, *M. alba* maintained biomass accumulation up to 25 mg·kg^-^¹ in the present study. This relatively high tolerance may be associated with its perennial growth habit and a stronger capacity for Cd retention in roots, which may reduce the transfer of toxic metals to aboveground tissues.

### 4.2 Photosynthetic suppression linked to nutrient imbalance and oxidative injury

Photosynthetic performance exhibited a clear threshold-dependent response to Cd exposure. Chlorophyll a, chlorophyll b, and total chlorophyll contents declined significantly in T4 and T5 (**Figure 2a**; P< 0.05), while Pmax increased slightly in T1 but decreased progressively in T3–T5 (**Table 1**). This pattern indicates a transition from temporary physiological compensation to functional impairment as Cd stress intensified (Li et al., 2025). Comparable declines in chlorophyll content and photosynthetic activity under Cd stress have been reported in several woody species (Haider et al., 2021; Li et al., 2025). The transient increase in Pmax at low Cd exposure observed here differs from responses reported in more sensitive species where such compensatory stimulation is absent. This discrepancy may reflect species-specific differences in redox regulation or in the capacity to maintain nutrient balance under metal stress.

Leaf nutrient status also changed markedly along the Cd gradient. Leaf N content declined from 5.06 g kg^-^¹ in CK to 2.97 g kg^-^¹ in T5, accompanied by significant reductions in P and K (**Table 2**). Because chlorophyll synthesis, Rubisco production, and ATP generation depend strongly on sufficient nutrient availability, such reductions are likely to constrain carbon assimilation (Haider et al., 2021). Mantel analysis (**Figure 10**) further showed that photosynthetic traits were more strongly correlated with root Cd content than with shoot Cd content, suggesting that belowground Cd accumulation may play an important role in disrupting plant metabolic processes (Behtash et al., 2025).

Oxidative stress appeared to further aggravate photosynthetic inhibition. The contents of H_2_O_2_ and MDA increased significantly in T4–T5 (**Figure 4**), indicating enhanced reactive oxygen species accumulation and membrane lipid peroxidation. At the same time, antioxidant enzyme activities declined at higher Cd levels, suggesting that the detoxification capacity of the antioxidant system was weakened under severe Cd stress (Zhang and Dong, 2025).

The temporary increase in Pmax observed at low Cd concentrations may be associated with redox-sensitive signaling triggered by mild ROS accumulation. Moderate ROS production can activate antioxidant defenses and influence phytohormonal regulation, thereby temporarily enhancing physiological activity (Su et al., 2022). Although these mechanisms were not directly examined in the present study, they may help explain the hormetic response observed under low Cd exposure.

Gas exchange measurements clearly indicated photosynthetic inhibition under elevated Cd concentrations. However, chlorophyll fluorescence parameters such as Fv/Fm and ETR were not measured, which limits a direct evaluation of PSII photochemical efficiency. Incorporating fluorescence measurements in future studies would help clarify the extent of photochemical damage induced by Cd stress (Chang et al., 2026).

The photosynthetic responses observed in *M. alba* under Cd exposure likely result from multiple interacting factors, including pigment degradation, nutrient limitation, oxidative damage, and redox-mediated regulatory adjustments.

### 4.3 Cd uptake and compartmentalization strategy

The patterns of BCF and TF indicate that Cd handling in *M. alba* varies with exposure level. Under low Cd exposure, BCF > 1 and TF > 1 indicate active Cd uptake accompanied by effective translocation to aboveground tissues. With increasing Cd concentrations, both indices declined, suggesting that Cd was increasingly retained in roots rather than transported to shoots (Li et al., 2012; Loix et al., 2017; Remelli et al., 2016).

Transmission electron microscopy further revealed structural features consistent with this shift in Cd allocation. Electron-dense deposits were observed along cell walls and within vesicles, indicating localized Cd accumulation. Mitochondrial deformation also became apparent under higher Cd exposure. Similar ultrastructural changes have been reported in other woody plants and are generally associated with detoxification processes such as cell wall immobilization, vacuolar sequestration, and apoplastic binding (Cannino et al., 2009; Xu et al., 2017).

The decline in TF combined with increased root Cd retention suggests a progressive transition from metal translocation to intracellular sequestration as Cd toxicity intensified. Root Cd content was more strongly associated with growth, metabolic, and defense-related traits, indicating that belowground Cd retention plays a key role in shaping plant responses to Cd stress. Comparable patterns have been reported in *Populus* and *Salix* species exposed to increasing Cd concentrations (Loix et al., 2017; Xu et al., 2017).

Unlike hyperaccumulator species that maintain efficient metal transport to shoots, *M. alba* appears to preferentially retain Cd in roots under elevated Cd exposure. Such a strategy is more consistent with phytostabilization than with active phytoextraction and may contribute to improved long-term stability of Cd-contaminated soils.

### 4.4 Stability of rhizosphere bacterial communities under Cd stress

Increasing Cd exposure did not lead to a clear reduction in bacterial α-diversity in the rhizosphere of *M. alba*. In many herbaceous systems, heavy metal stress is often accompanied by a pronounced decline in microbial diversity (Luo et al., 2017). The relatively stable diversity observed in this study may reflect the buffering capacity of woody perennial root systems and their long-term interactions with rhizosphere microorganisms. Similar patterns of compositional stability have been reported in several forest species exposed to chronic metal contamination, suggesting that perennial hosts may maintain relatively resilient microbial communities under environmental stress.

Although overall diversity remained relatively constant, noticeable shifts occurred in community composition at the genus level. In particular, *Pseudomonas*, *Bacillus*, and *Acinetobacter* increased in relative abundance along the Cd gradient. These genera are widely reported to possess metal tolerance traits, including metal efflux systems, exopolysaccharide production, and metal-chelating mechanisms that facilitate survival under heavy metal stress (Nie et al., 2023; Wu et al., 2022; Yin et al., 2024).

Such compositional changes may indicate adjustments in the functional potential of the rhizosphere microbiome. However, the present interpretation relies on taxonomic profiles and predictive functional inference based on PICRUSt2 rather than direct functional measurements. Therefore, the observed shifts should be regarded as indicative rather than definitive evidence of functional change.

Rather than inducing a complete restructuring of the microbial community, Cd exposure appears to act mainly as a selective pressure that modifies the relative abundance of specific taxa while maintaining the dominance of major bacterial phyla. This pattern is consistent with the perennial growth habit of woody plants and the stability often associated with long-term plant–microbe interactions in forest soils.

The stability observed at higher taxonomic levels also corresponds with the strong role of root Cd retention revealed in the multivariate analysis. Together, these results suggest that coordinated plant–microbe interactions may contribute to maintaining rhizosphere stability under Cd stress.

### 4.5 Implications for phytoremediation

Compared with other woody species such as *Populus* and *Salix*, which often experience substantial physiological disturbance under moderate Cd loads, *M. alba* maintained relatively stable growth, photosynthetic activity, osmotic regulation, and rhizosphere stability at Cd concentrations up to 25 mg·kg^-^¹. Although this species does not exhibit the extreme accumulation capacity characteristic of specialized Cd hyperaccumulators, its tolerance to Cd stress, ability to retain metals in roots, and large perennial biomass make it well suited for long-term phytostabilization (Zulfiqar et al., 2022).

The responses observed in this study are broadly consistent with earlier reports describing growth inhibition and oxidative imbalance in woody plants exposed to increasing Cd concentrations (Hu et al., 2025; Zeng et al., 2020). Similarly, biomass production and photosynthetic performance declined once Cd concentrations exceeded a certain threshold (Haider et al., 2021). However, a notable feature of the present study is the clear dose-dependent shift from metal translocation to enhanced root retention. Multivariate analyses identified root Cd accumulation as a key factor associated with the observed physiological responses. Such differences among studies may reflect variation in species-specific metal compartmentalization capacity, root apoplastic binding, or the Cd exposure gradients applied in experimental systems.

By integrating physiological responses, metal allocation patterns, and rhizosphere community characteristics within a unified analytical framework, this study provides a system-level perspective on Cd tolerance in perennial woody plants (Kazmi et al., 2025). The coordinated responses observed in *M. alba* suggest a tolerance strategy involving root-based detoxification, regulation of shoot physiological processes, and buffering effects within the rhizosphere (Behtash et al., 2025). These characteristics indicate that *M. alba* may represent a promising species for the remediation of mildly to moderately Cd-contaminated soils through gradual metal stabilization and long-term ecosystem recovery.

### 4.6 Multivariate analysis

Multivariate analyses revealed clear patterns in plant physiological responses to Cd stress. PCA separated growth- and metabolism-related traits from stress-associated variables along the main ordination axis, indicating that Cd exposure induces coordinated adjustments across multiple functional processes rather than isolated changes in individual traits. The clustering of biomass, photosynthetic pigments, and nutrient-related variables along the same axis further suggests that these processes are closely linked and respond jointly to increasing Cd levels (Nocito et al., 2011).

Mantel analysis further clarified the relationships underlying this multivariate structure. Root Cd content showed stronger and more frequent correlations with physiological traits than shoot Cd content, highlighting the importance of belowground Cd accumulation in shaping whole-plant responses. In particular, growth performance, nutrient status, and antioxidant regulation were strongly associated with root Cd levels, suggesting that Cd retained in roots exerts a greater influence on plant physiological regulation than Cd transported to aboveground tissues (Kosakivska et al., 2021; Podar and Maathuis, 2022).

The consistency between the PCA results and Mantel correlations indicates a coordinated response pattern under Cd stress. Root Cd accumulation appears to act as an upstream factor linking growth inhibition, metabolic adjustment, and defense activation. Through this mechanism, belowground Cd retention contributes to the overall organization of physiological responses and may support plant tolerance under metal exposure. Identifying root Cd c+ontent as a dominant structuring variable extends previous single-trait analyses by emphasizing coordinated multivariate responses. While earlier studies often focused on individual physiological indicators, the integrative approach used here reveals a hierarchical organization of tolerance mechanisms centered on root-level metal retention.

**Figure 11:**
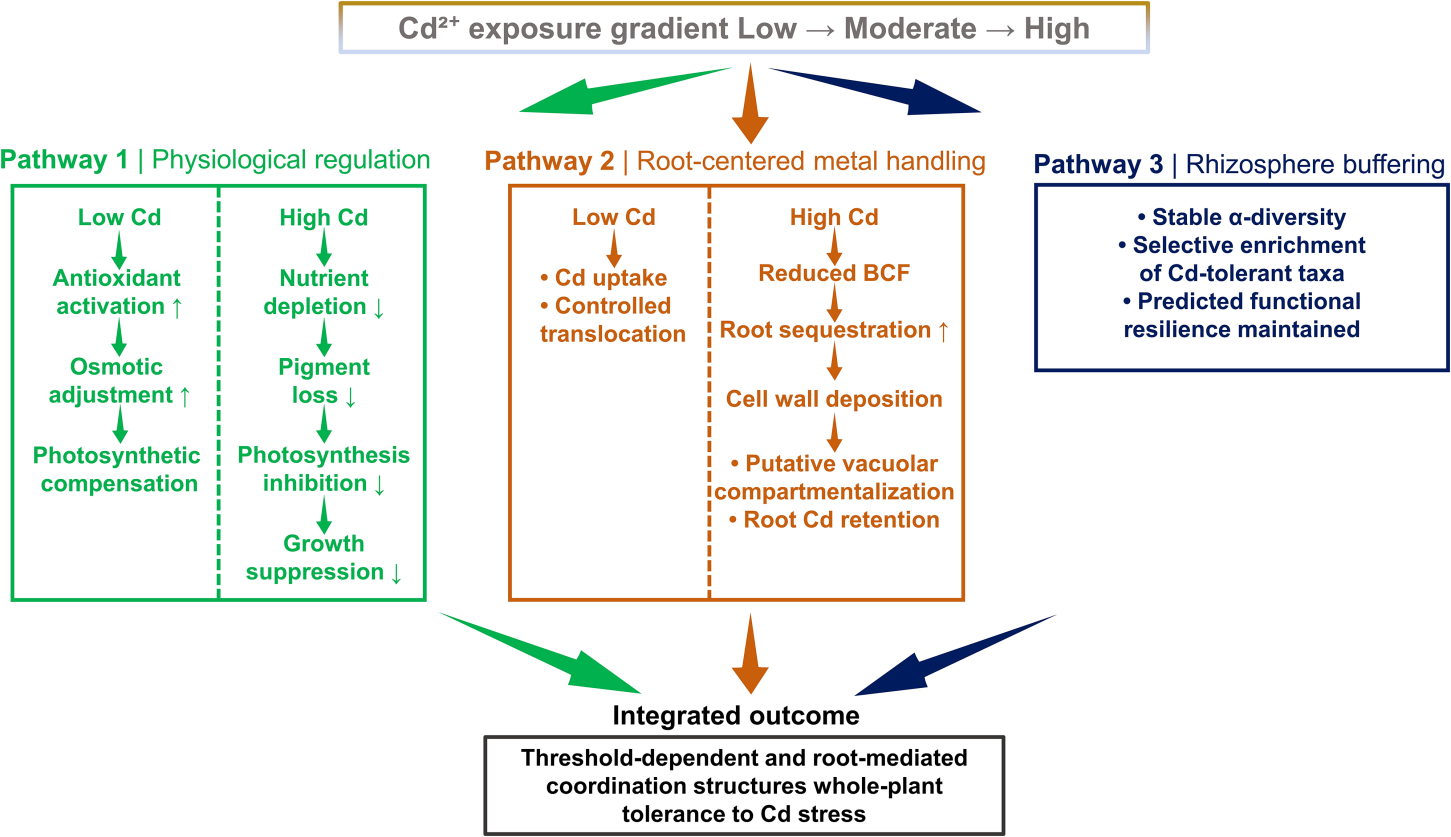
Integrated tolerance framework of *M. alba* under Cd stress. The schematic summarizes the threshold-dependent response of M. alba along a Cd gradient. Low Cd induces compensatory antioxidant, osmotic, and photosynthetic adjustments, whereas higher Cd levels lead to nutrient depletion, growth inhibition, and enhanced root sequestration, including cell wall deposition and putative vacuolar compartmentalization. Rhizosphere bacterial diversity remains stable with selective enrichment of Cd-tolerant taxa. Root Cd retention emerges as a central structuring factor associated with coordinated whole-plant tolerance.

Based on the combined physiological, ultrastructural, and multivariate evidence, a conceptual framework summarizing the coordinated tolerance strategy of *M. alba* under Cd stress is proposed (**Figure 11**).

### 4.7 Limitations and future research directions

Despite the insights obtained in this study, several limitations should be acknowledged when interpreting the results. The experiment was conducted as a pot trial under controlled conditions using a uniform soil matrix and defined Cd additions. Such conditions simplify many environmental variables and therefore cannot fully represent the complexity of field soils. In natural systems, factors such as Cd speciation, soil organic matter content, and interactions with competing ions can strongly influence metal bioavailability and plant uptake processes (Mo et al., 2021). In addition, soil Cd concentrations were not re-measured at the end of the experiment. This limitation restricts the evaluation of Cd redistribution among exchangeable, bound, and insoluble fractions and prevents a precise assessment of net Cd removal or stabilization at the soil level.

Another limitation relates to the analysis of microbial functions. The present study relied on 16S rRNA amplicon sequencing combined with predictive metagenomic inference, which reflects potential functional capacity rather than direct microbial activity. More comprehensive approaches, such as shotgun metagenomics, metatranscriptomics, or enzyme activity assays, would provide stronger evidence for the functional roles of Cd-tolerant taxa in metal detoxification and nutrient cycling. In addition, the experiment covered only a single growing season and focused on young saplings. Consequently, long-term patterns of Cd accumulation, physiological acclimation, and biomass–metal relationships across multiple growth cycles remain uncertain (Mahar et al., 2016).

Further research is required to better clarify the mechanisms of Cd tolerance and the remediation potential of *M. alba*. Future studies could further investigate Cd speciation and bioavailability at different developmental stages using advanced techniques such as X-ray absorption spectroscopy or synchrotron-based analyses (Mo et al., 2021). Integrating metabolomic, ionomic, and transporter-level analyses would also improve understanding of the physiological mechanisms governing Cd uptake, sequestration, and retention (Yu et al., 2024). In the rhizosphere, combining metagenomic approaches with culture-based techniques or enzyme activity measurements would allow more direct evaluation of microbial contributions to Cd immobilization and nutrient transformation (Jing et al., 2007). Long-term field experiments will also be necessary to assess remediation efficiency, biomass turnover, and soil Cd dynamics under realistic environmental conditions (Subašić et al., 2022).

It should also be noted that ultrastructural observations were not accompanied by elemental microanalysis. Techniques such as energy-dispersive X-ray spectroscopy (EDS) could directly verify Cd localization within the electron-dense deposits observed in cell walls and vesicles. Without such verification, the interpretation of these structures as Cd accumulation sites remains tentative (Kuang et al., 2023).

Finally, because soil Cd concentrations were not re-quantified at the end of the experiment, a complete Cd mass balance within the soil–plant system could not be established. Incorporating soil Cd speciation analysis together with mass balance approaches in future studies would provide a more accurate evaluation of remediation efficiency and stabilization potential (Yang et al., 2022).

## 5 Conclusions

The present study shows that M. alba responds to cadmium (Cd) stress through coordinated adjustments in plant physiology, cellular detoxification processes, and rhizosphere microbial communities. Increasing Cd concentrations progressively reduced plant growth, nutrient assimilation, pigment synthesis, and photosynthetic performance, while simultaneously intensifying oxidative stress and membrane damage. Under low Cd exposure, however, plants showed a hormetic response characterized by enhanced antioxidant activity, osmotic regulation, and a temporary increase in photosynthetic capacity. Patterns of Cd bioaccumulation and translocation, together with ultrastructural observations, indicate a dose-dependent shift in metal handling from shoot translocation toward root retention and intracellular compartmentalization at higher Cd levels. At the same time, the relatively stable bacterial α-diversity and the enrichment of several putatively Cd-tolerant taxa suggest that Cd exposure alters community composition without causing a collapse of rhizosphere microbial structure, although further functional validation is still required. Collectively, the physiological, ultrastructural, and microbial patterns observed in this study suggest that M. alba has a strong capacity to tolerate Cd exposure. These characteristics suggest that this species may be suitable for the remediation of mildly to moderately Cd-contaminated soils through long-term stabilization processes.

## Data Availability

The raw sequencing data have been deposited in the NCBI Sequence Read Archive (SRA) under BioProject accession number PRJNA1439574. Other data supporting the findings of this study are available from the corresponding author upon reasonable request.
